# Umbilical hernia repair post umbilical cord graft closure of gastroschisis: A cohort study

**DOI:** 10.1016/j.ijscr.2022.107175

**Published:** 2022-05-11

**Authors:** Heba Taher, Hajar Khalil, Saad Ahmed, Mostafa Gad, Belal Elezaby, Abdelazez Magdy, Khaled S Abdullateef

**Affiliations:** a Pediatric Surgery, Cairo University, Egypt; b Cairo University, Egypt; c Pediatric Surgery, Azhar University, Asyiut, Egypt

**Keywords:** Umbilical cord graft, Umbilical hernia, Gastroschisis

## Abstract

**Introduction:**

Gastroschisis a common congenital anomaly in the anterior abdominal wall, the bowel is present outside the abdominal cavity, completely devoid of any coverings, management of gastroschisis involves umbilical cord graft coverage of the defect after bowel reduction when there are concerns about compartmental syndrome, this is a widely used technique but there are few reports about the incidence umbilical hernia development after this technique and need for future repair of the defect.

**Presentation of cases:**

We had 8 patients with simple gastroschisis who had umbilical cord graft coverage of the defect at birth between 2017 and 2020, we present 4 patients who had the cord graft without cutting of rectus fascia, 2 patients resolved spontaneously and 2 developed an umbilical hernia requiring repair.

**Discussion:**

Umbilical cord graft has been reported in several studies, in those studies the authors reported the spontaneous closure of the defect and some reported that incising the rectus fascia will contribute to development of the umbilical hernia, in our series the rectus fascia was preserved yet 2 patients developed umbilical hernia.

**Conclusion:**

Pediatric surgeons should look out for umbilical hernia in patients who had umbilical cord graft repair of gastroschisis defect and closure should be carried out by an experienced surgeon.

## Highlights

• Umbilical cord graft is used in closure of gastroschisis defect.
• Umbilical cord graft used when there are concerns about compartment pressure.
• Umbilical hernia can develop following this technique.
• Incision of rectus fascia increases the incidence of umbilical hernia.
• Umbilical hernia repair can be challenging due to adhesions.

## 1 Introduction

Gastroschisis is the most common congenital anomaly in the anterior abdominal wall [1]. It occurs when the bowel is present outside the abdominal cavity, completely devoid of any coverings through a defect that is usually to the right of the umbilicus in the anterior abdominal wall - being exposed to amniotic fluid in the uterus. Its incidence increased in the past decade to reach 1 in 1953 live births worldwide, with an increased risk in young mothers [2]. The exact aetiology is unknown. Gastroschisis can be identified by routine ultrasonography in the prenatal visits starting from the 14th week of gestation [3]. It is important to differentiate it from an omphalocele- which is the herniation of the bowel through the umbilicus covered by the peritoneum, amniotic membrane, and Wharton's jelly respectively- and to plan delivery in a qualified hospital that can manage gastroschisis [4].

Management principles of gastroschisis are reducing the bowel into the abdominal cavity with an adequate intra-abdominal pressure, to avoid compartmental syndrome, and subsequently closing the defect. Options include primary repair with fascial and skin suturing as well as gradual reduction using silo when a high intra-abdominal pressure is recorded followed by surgical repair.

In 1998, Bianchi and Dickson described, for the first time, another method entailing delayed reduction without general anesthesia and closure using the umbilical cord as a graft in 14 cases by suturing it to the rectus sheath [5]. In another study, an attempt was made to close the defect without sutures to allow for spontaneous healing that resulted in a closed defect and a normally positioned umbilicus [6].

In this study, we present 4 cases born with simple gastroschisis where an umbilical cord graft was used for primary closure of the defect without incision of the fascia and the likelihood of developing an umbilical hernia that either closes spontaneously or requires surgical repair.

This work has been reported in accordance with the PROCESS 2020 guidelines [7].

## 2 Methods

During the period between 2017 and 2020 at Cairo university hospital, a tertiary pediatric surgery center, we had 8 patients with simple gastroschisis and had no other intestinal pathologies such as volvulus, perforation and atresia. They underwent reduction and closure of the defect using an umbilical cord graft.

This is a retrospective cohort study in which we included four patients who had gastroschisis repair right after birth using umbilical cord graft without cutting of the fascia during the procedure and were followed up to check if their umbilical defect closed spontaneously within a 2 year period and if they needed repair of umbilical hernia. The 4 Patients who had fascial extension to facilitate reduction were excluded from this study as this manoeuvre is reported to interfere with spontaneous closure of the umbilical defect [6]. However 2 of them developed an umbilical hernia and remaining 2 couldn't be contacted. Since this was a retrospective study no ethical approval was required to carry out the procedure. On the other hand parental consent was obtained before any intervention was carried out to the child. This study is on research registry with research register unique identifying number researchregistry7862.

## 3 Results

Of the four patients who had umbilical cord graft, two patients had spontaneous closure of the umbilical defect while the other two had persistent defects, one of which already underwent a hernia repair and the second has a pending operation (Table 1).

**Table 1:** Showing important patient and maternal indicators and outcome.

Cases	General condition	Bowel condition	Mother's age	Hour of closure (post-operatively)	Need for hernia repair
Case 1	Healthy	Normal	28	1	No need
Case 2	Healthy	Oedematous	32	6	Yes
Case 3	Healthy	oedematous	16	3	No need
Case 4	Healthy	oedematous	16	6	Yes

## 4 Case presentation

### 4.1 Case 1

**Fig. 1:**
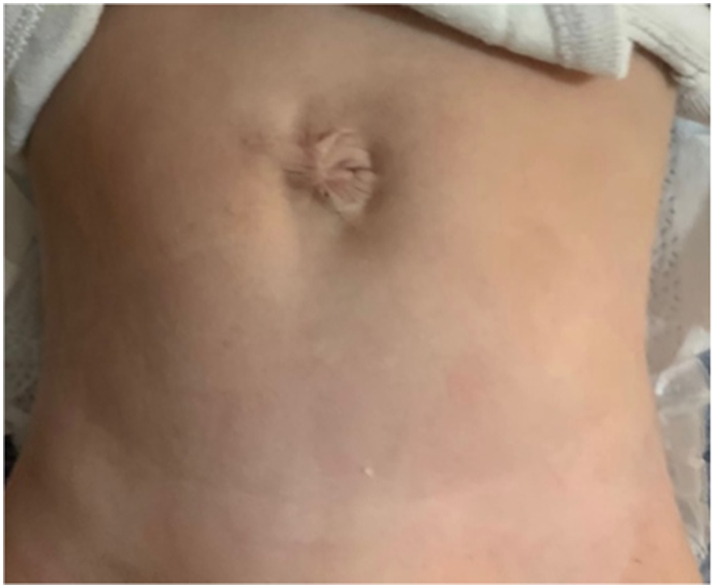
Patient one 8 months post gastroschisis closure using umbilical cord graft.

A male patient presenting with gastroschisis that was diagnosed on prenatal ultrasonography and was born full-term through normal vaginal delivery. The mother was 28 years old, and this baby was her second child. The baby was healthy at birth and the bowel was neither oedematous nor associated with other anomalies. We attempted repair using the umbilical cord at 1 h post-delivery and the defect closed spontaneously without the need for an elective surgery (Fig. 1).

### 4.2 Case 2

**Fig. 2:**
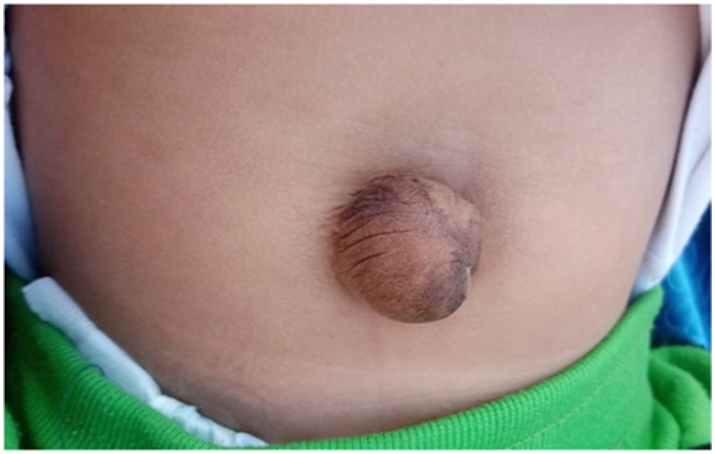
One year post umbilical graft patient has umbilical hernia.

A 32-year-old mother gave birth to a male baby born with gastroschisis, at full-term, after a normal vaginal delivery. The bowel was oedematous at presentation and the repair was delayed 6 h due to the timing of presentation and resuscitative measures. We used the umbilical cord grafting technique and the child developed umbilical hernia (Fig. 2). The patient is now 2 years old with a persistent hernia that is scheduled for elective closure.

### 4.3 Case 3

**Fig. 3:**
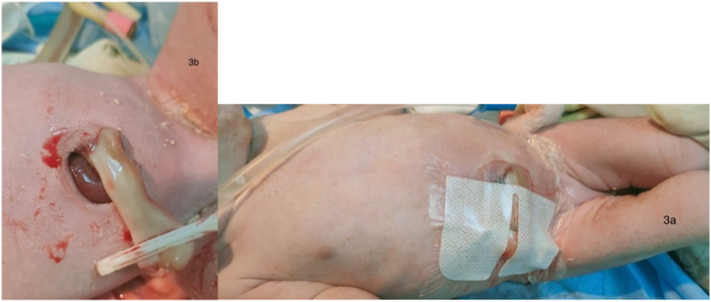
Had umbilical cord graft sutureless technique.

Another male patient born at full-term following an uncomplicated caesarean delivery to 16 year-old mother with positive consanguinity. Baby had gastroschisis that was diagnosed on prenatal ultrasound. The bowel was oedematous at first presentation and the defect was closed at 3 h post-delivery using the umbilical cord as a graft (Fig. 3). The defect then healed completely without the need for another surgery.

### 4.4 Case 4

**Fig. 4:**
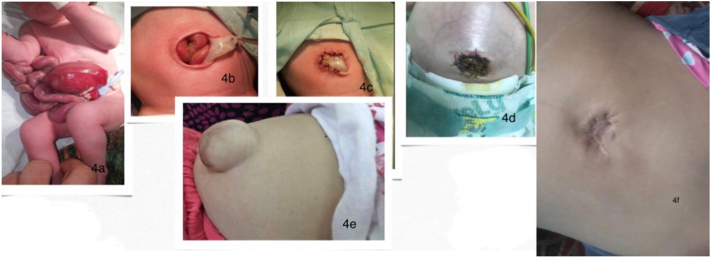
a bowel appearance 6 h after birth; c: immediate post-operative appearance, d: one week after umbilical graft placement, e: 3 months after the graft placement; f post umbilical hernia repair at age of 3 years.

A 16 year-old mother presented with her baby girl 3 h after having an obstructed vaginal delivery. The mother gave a history of trauma following falling. Not feeling the baby moving for a couple of days. The baby had gastroschisis with an oedematous bowel (Fig. 4a) and the operation was done at 6 h post-delivery (Fig. 4b-c). The baby also developed an umbilical hernia (Fig. 4e) which we observed conservatively for spontaneous closure. At 2.5 years, due to failure of closure, we operated on the baby to repair the hernia (Fig. 4f).

All patients were admitted to neonatal surgical ICU to receive preoperative resuscitation starting by warming and avoiding heat losses by keeping the infant on an external heating device or a Radiant warmer, insertion of a central line, urinary catheter and a nasogastric tube. Hydration was done using IV fluid normal saline until infant's urine output normalises and/or blood gases indicate normal acid-base balance. Albumin and plasma can be used to avoid volume overload after several IV boluses. Broad spectrum IV antibiotics such as gentamicin and ampicillin are prescribed as well.

The initial procedures of bowel reduction and grafting were carried out by an assistant lecturer equivalent to 4th year surgical training under supervision of a consultant. While umbilical hernia repair was performed by the consultant.

## 5 Discussion

Primary closure of gastroschisis has long been done through the approximation of skin flaps surrounding the defect to accommodate the protruding bowel inside the abdominal cavity. In 1967, Schuster performed staged reduction of the bowel for the first time in cases where primary closure was not appropriate. He used Teflon sheets and skin flaps to close the defect and opened the abdomen repeatedly to reduce small portions of the bowel until all has been reduced [8]. This later developed into the use of Silastic silo in 1997, a widely used technique for staged reduction of gastroschisis nowadays [9].

In 1998, the idea of umbilical cord grafting was introduced, and studies have been done to explore and identify its advantages. This method utilises the new-born's ability at rapid healing and the circumferential closure of the umbilical defect by cicatrisation. It operates on early reduction to avoid the bowel becoming inflamed and oedematous which adds unwanted complications, provided the bowel is not oedematous on delivery. We observed that using this technique resulted in a more cosmetically pleasing appearance of the umbilicus which becomes centralised, owing to the process of periumbilical circumferential cicatrisation with no scarring [5], [6].

Studies have also described the advantages of using this procedure to avoid compartment syndrome. Other methods mentioned before require suturing of the fascia and skin which limits the intra-abdominal space and may result in a significant rise in the intra-adnominal pressure, risking the development of acute compartment syndrome [6]. Acute compartment syndrome is a constant rise in the intra-abdominal pressure (IAP) above 10 mmHg that leads to ischemia of the abdominal organs quickly, escalating to necrosis if emergency surgery is not done [10]. Studies show that closure of the defect using the umbilical cord does not result in a rise in the abdominal pressure even when the space is limited, which allowed the babies to be fed at an earlier time; improving the overall health and prognosis [6].

**Fig. 5:**
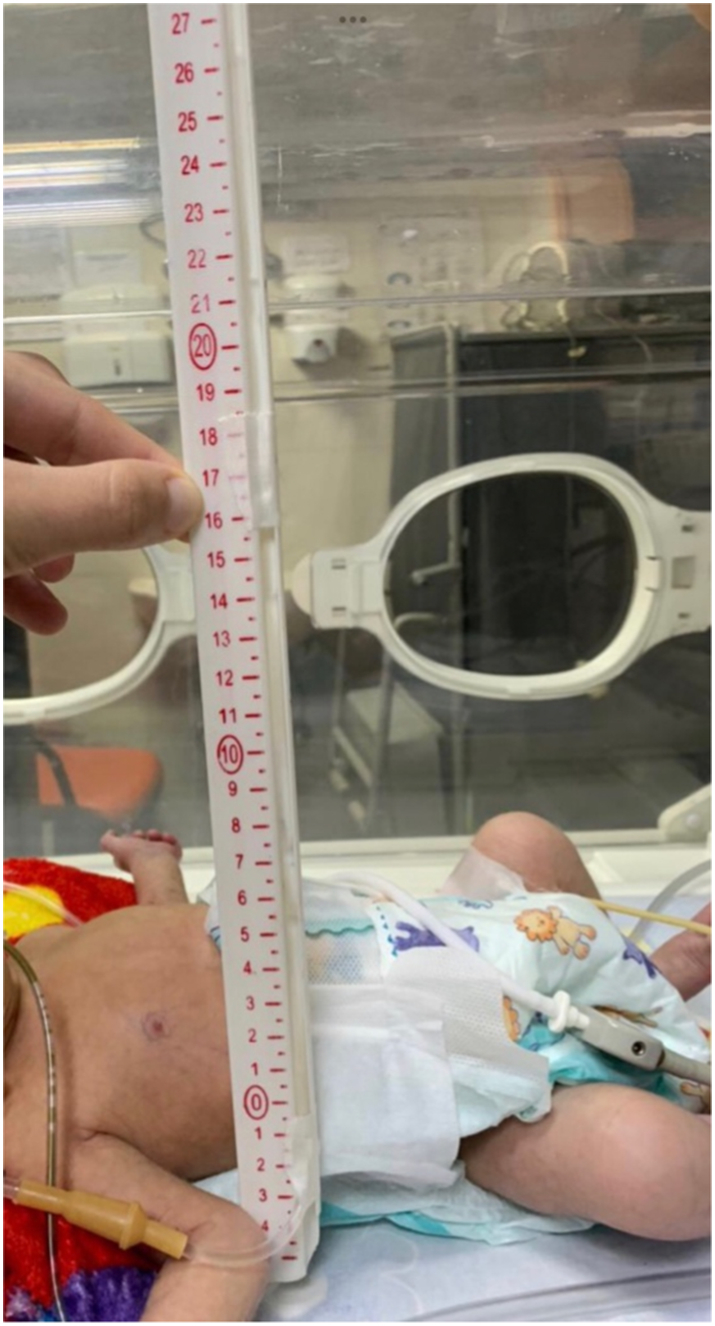
position of the patient during central line measurement.

In a recent prospective study involving 20 patients with gastroschisis, a selection criterion was used to decide on the method of closure based on the IAP through several parameters. Firstly, the intravesical pressure (IVP) which utilises a system filled with saline inserted into the bladder. The umbilical cord was used when the readings where equivalent to >15 mmHg. Secondly, since acute compartment syndrome is essentially bowel ischemia, abdominal perfusion pressure (APP) was used as a better indicator for the bowel blood supply where values <50 mmHg were closed using an umbilical cord graft. APP is measured by subtracting the IAP from the Mean Arterial Pressure (MAP).Thirdly, the peak inspiratory pressure which is the pressure used to inflate the alveoli by the ventilator, a cut off value of 24 cmH_2_O determined the use of the umbilical cord. Lastly, following the insertion of a central line (Fig. 5), the central venous pressure was measured and values >15 cmH_2_O indicated the use of the umbilical cord graft (Fig. 2) [10].

Even though this method seems foul-proof, one of the outcomes to look out for is the development of an umbilical hernia after complete healing, which was described in a few studies [5], [6], [10], [11] particularly after cutting the rectus fascia [6], as well as shown in our cases; though in our patients the rectus fascia was not breeched. We followed up a cohort of four patients who had umbilical cord graft without cutting of the of rectus fascia. [6] to observe spontaneous closure of the hernia that usually occurs within 2 years. All 14 cases in the Bianchi and Dickson study closed spontaneously [5]. In another study, involving 10 children, only one patient required umbilical herniorrhaphy after failure of spontaneous closure [6]. In our case series, 2 patients did not require surgical intervention, 2 developed umbilical hernia despite not creating a facial incision in the rectus sheath. One is yet to be surgically repaired, and one has been closed electively at 2.5 years- which we found was harder than usual. Due to the manipulation of the bowel with the process of reduction, dense adhesions develop in the abdominal cavity between the bowel and the umbilical scar, making the umbilical hernia repair more challenging. It needs the surgical experience of a senior registrar or even a consultant.

There is no direct indicator to tell how the hernia will behave following the repair of the gastroschisis defect and whether it is more likely to need an intervention or not. A long term study with a bigger sample size is needed to look into this outcome and determine the indicators of spontaneous closure. Points to be considered in future studies are accurate measurements of the defect size at closure.

Moreover, In the future artificial intelligence might play a role in gastroschisis management by creating algorithms based on data generated from central lines and ventilators and suggested best surgical approach for the neonate [12], [13], [14].

## 6 Conclusion

The process of managing gastroschisis starts with the correct prenatal diagnosis and ends with proper treatment to get the best overall results. The babies' general condition should be assessed with stabilisation methods performed if needed. The bowel should be inspected for oedema as well as other associated anomalies, then the role of different treatment strategies should be considered. Postoperative care and nutritional support is very crucial [15]. Even though science is advocating for gastroschisis repair using umbilical cord graft for its impressive end results, we should note that the developing umbilical hernia is not to be taken lightly as its surgical repair is done with caution due to bowel adhesions to anterior abdominal wall. We also advocate umbilical cord grafts for patients presenting relatively late 4–5 h post birth when oedema sets in the bowel making facial and skin closure more challenging and according to objective measurement criteria [10].

## Funding

There is no funding for this work.

## Ethical approval

No ethical approval was needed for this study as it is retroscpective.

## Consent

Parental consent was obtained since patients are minors.

## Author contribution

Heba Taher drafting operating and data collection.

Hajar Khalil: drafting following up patients assistant.

Saad Ahmed: operating.

Belal Ezaby: operating and data collection.

Mostafa Gad: operating.

Abdulaziz Magdy: operating and assisting.

Khaled S Abdullateef: operating.

## Registration of research studies

1. Name of the registry: research registry.
2. Unique identifying number or registration ID: researchregistry7862.
3. Hyperlink to your specific registration (must be publicly accessible and will be checked): https://www.researchregistry.com/registernow#home/registrationdetails/626e97b39051b7001ecd0794/.

## Guarantor

Heba Taher.

## Declaration of competing interest

Authors declare no conflict of interest.
